# Identification of Autophagy in the Pine Wood Nematode *Bursaphelenchus xylophilus* and the Molecular Characterization and Functional Analysis of Two Novel Autophagy-Related Genes, *BxATG1* and *BxATG8*

**DOI:** 10.3390/ijms17030279

**Published:** 2016-03-03

**Authors:** Li-Na Deng, Xiao-Qin Wu, Jian-Ren Ye, Qi Xue

**Affiliations:** 1 Co-Innovation Center for Sustainable Forestry in Southern China, College of Forestry, Nanjing Forestry University, Nanjing 210037, Jiangsu, China; denglina121@163.com (L.-N.D.); jrye@njfu.edu.cn (J.-R.Y.); xq2159@163.com (Q.X.); 2 Jiangsu Key Laboratory for Prevention and Management of Invasive Species, Nanjing Forestry University, Nanjing 210037, Jiangsu, China; 3 Yancheng Institute of Technology, School of Ocean and Biological Engineering, Yancheng 224051, Jiangsu, China

**Keywords:** *Bursaphelenchus xylophilus*, autophagy, transmission electron microscopy, autophagy-related genes, *in situ* hybridization, RNA interference

## Abstract

The pine wood nematode, *Bursaphelenchus xylophilus*, causes huge economic losses in pine forests, has a complex life cycle, and shows the remarkable ability to survive under unfavorable and changing environmental conditions. This ability may be related to autophagy, which is still poorly understood in *B. xylophilus* and no autophagy-related genes have been previously characterized. In this study, transmission electron microscopy was used to confirm that autophagy exists in *B. xylophilus*. The full-length cDNAs of *BxATG1* and *BxATG8* were first cloned from *B. xylophilus*, and *BxATG1* and *BxATG8* were characterized using bioinformatics methods. The expression pattern of the autophagy marker *BxATG8* was investigated using *in situ* hybridization (ISH). *BxATG8* was expressed in esophageal gland and hypodermal seam cells. We tested the effects of RNA interference (RNAi) on *BxATG1* and *BxATG8*. The results revealed that *BxATG1* and *BxATG8* were likely associated with propagation of nematodes on fungal mats. This study confirmed the molecular characterization and functions of *BxATG1* and *BxATG8* in *B. xylophilus* and provided fundamental information between autophagy and *B. xylophilus*.

## 1. Introduction

*Bursaphelenchus xylophilus* (Steiner & Buhrer) Nickle is the pine wood nematode that is the causal agent of pine wilt disease (PWD), which results in large economic losses [1]. *B. xylophilus* is native to North America [2] but has been introduced to, and spread throughout, many parts of the world, including Asia and Europe, including Japan, China, South Korea and Portugal [3,4,5]. It has become a severe threat to pine forests worldwide [4,5,6]. At present, there are many different hypotheses to explain the pathogenesis of PWD, such as the cellulose (which suggests that the destruction of pine cells is triggered by cell wall-degrading enzymes, such as cellulose), phytotoxin and terpenoid hypotheses [7,8,9], but the pathogenic mechanism of *B. xylophilus* remains unknown.

*B. xylophilus* is a pathogenic nematode with a complex life cycle and occurs in two phases—dispersal and propagation [10]. Under unfavorable environmental conditions, such as limited food and cooler temperatures, the second-stage propagative juvenile molts into the third-stage dispersal juvenile, then they molt into specialized dispersal-stage dauer juvenile [10,11]. *B. xylophilus* shows a remarkable adaptability to changing environmental conditions, but the mechanism behind this adaptability is still not well understood. Under conditions of high population density, limited food or increased temperature, *Caenorhabditis elegans* nematodes can induce the process of autophagy [12,13]. *C. elegans* is used as a model organism and provides a wealth of information for research on other nematodes. Does the process of autophagy exist in *B. xylophilus*? Does autophagy assist the nematodes’ responses to various changing environmental conditions and allow them to invade pine trees successfully?

Autophagy exists widely in eukaryotic organisms and is an evolutionarily conserved process [14,15], in which protein and organelles are sequestered within double membrane vesicles that deliver the contents to the lysosome/vacuole for degradation and the recycling of the resultant macromolecules [16]. Autophagy is the major cellular pathway for the degradation of long-lived proteins and cytoplasmic organelles. It involves the rearrangement of subcellular membranes to sequester cargo for delivery to the lysosome where the sequestered material is degraded and recycled [14]. It has a greater variety of physiological and pathophysiological roles than expected, such as starvation adaptation, intracellular clearance, development, anti-aging, degradation of invading bacteria and cell death [15,17,18]. Recently, the role of autophagy was confirmed in pathogens and insect pests, such as *Magnaporthe grisea*, *Tenebrio molitor* and *Rhipicephalus* (*Boophilus*) *microplus* [19,20,21,22], and it plays an important role in their growth, development, reproduction and pathogenicity. Whether the autophagy of *B. xylophilus* is associated with adaptability to changing environmental conditions, vitality, reproduction, invasiveness and pathogenicity is still unknown. Therefore, insights into the characteristics of autophagy and its functions in *B. xylophilus* may help in better understanding the biological adaptation and pathogenic mechanisms.

An objective of this study is to show that autophagy exists in *B. xylophilus* using transmission electron microscopy (TEM). TEM is a very reliable approach to analyzing and quantifying autophagic compartments. TEM allows the visualization of every step of the autophagic pathway [23]. The genes responsible for autophagy were first characterized in the yeast *Saccharomyces cerevisiae* [24]. Out of the many *ATG* gene nucleotide sequences of eukaryotic organisms, from yeast to mammals [15,21,24], we were particularly interested in *ATG1* and *ATG8* because the *ATG1* product plays an essential role in the regulation of autophagy [19,25], and the *ATG8* product performs an important role in the formation of double-membrane autophagosomes, a central step in the intracellular degradation pathway of autophagy, which is routinely used as a marker when studying autophagy [20,26]. Our study sought to clone two novel autophagy-related genes, *ATG1* and *ATG8*, in *B. xylophilus*, named *BxATG1* and *BxATG8*, respectively. The serine/threonine kinase ATG1 plays an essential role in stimulating autophagy, however, autophagy is a process, and the localization of *BxATG8* allows us to track autophagosomes from their initiation in the cytoplasm to their degradation inside the vacuole. Thus, we assessed the functions of autophagy in *B. xylophilus* using *in situ* hybridization (ISH) to investigate the localization of *BxATG8* expression. RNA interference (RNAi) was used to assess the functions of *BxATG1* and *BxATG8*. The role of the autophagy genes *BxATG1* and *BxATG8* in development and reproduction through the turnover of organelles and proteins forms an attractive topic for research and is the focus of this paper.

## 2. Results

### 2.1. Qualitative Identification of Autophagy in B. xylophilus by Transmission Electron Microscopy (TEM)

**Figure 1:**
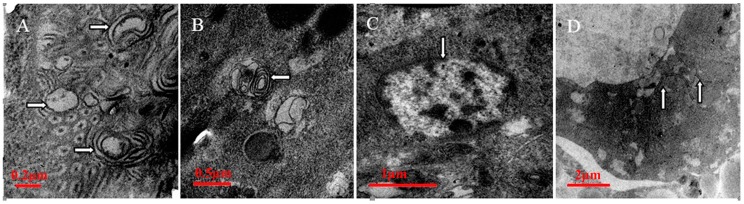
Autophagy in the cells of the highly virulent strain AmA3 of *Bursaphelenchus xylophilus* after starvation was induced for 12 h (**A**,**B**); 24 h (**C**); and 36 h (**D**), with autophagic bodies (right arrows), autophagosomes (left arrows), autolysosomes (down arrows) and vesicle breakdown (up arrows) Scale bars: (**A**) 0.2 µm; (**B**) 0.5 µm; (**C**) 1 µm; and (**D**) 2 µm.

TEM was used to identify autophagy in *B. xylophilus*. Many types of autophagic vacuoles of *B. xylophilus* are shown in Figure 1. The initial form is an autophagic body delineated by double-membranes (Figure 1A). Autophagosomes, which are characteristic features of the sequestering membrane are liable to be split into myelinated structures (Figure 1B). Autophagosomes that fuse with lysosomes degrade the content resulting in only clumps of the dense material (Figure 1C). The breakdown of the vesicle membrane allows the degradation of its cargo and the eventual recycling of the amino acids (Figure 1D). The TEM observations showed that the process of autophagy exists in *B. xylophilus*.

### 2.2. Autophagy-Related Gene Homologues in B. xylophilus

**Figure 2:**
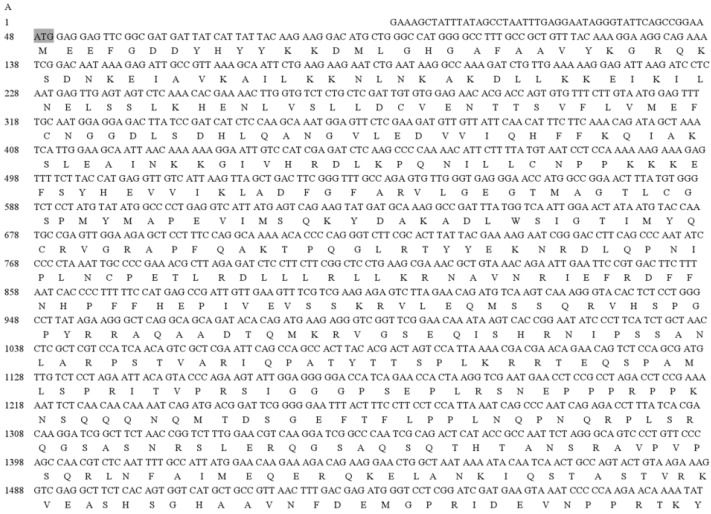

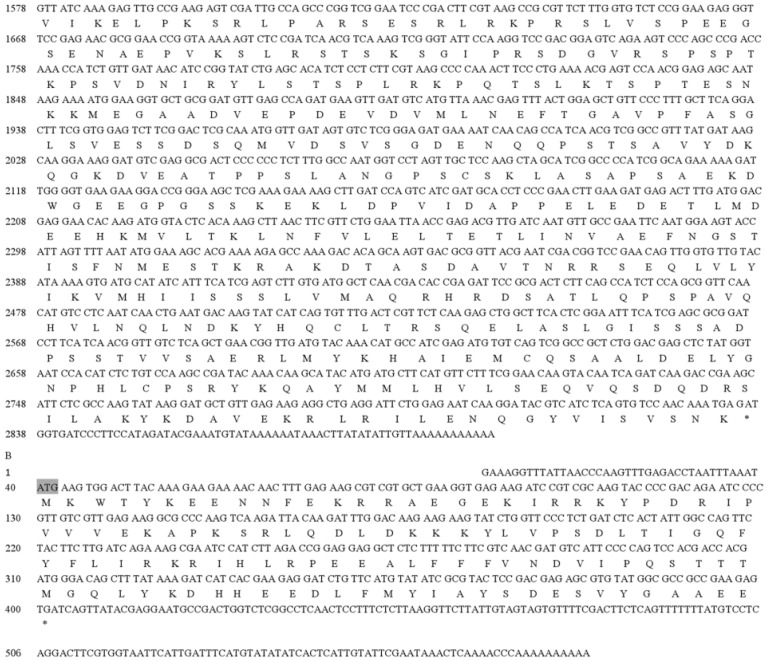
Full-length cDNA sequences and deduced amino acid sequence of *BxATG1* (**A**) and *BxATG8* (**B**) from *B. xylophilus*. Note: The initiation codons are shown with a dark background, and asterisks indicate the stop codons.

A homology-based cloning approach was used to obtain partial sequences of the *ATG1* and *ATG8* homologous sequences from *B. xylophilus*. The 3′ RACE and 5′ RACE PCR amplifications were used to obtain the full-length cDNA sequences of *BxATG1* and *BxATG8* from *B. xylophilus*. The flanking region of the *BxATG1* cDNA is 2901 bps, has an open reading frame (ORF) at position 48–2834 and encodes a 928 amino acid polypeptide (Figure 2A). The flanking region of *BxATG8* cDNA is 581 bps, has an ORF at position 40–402 and encodes a 120 amino acid polypeptide (Figure 2B).

### 2.3. In Situ Hybridization (ISH) for the Localization of BxATG8 in B. xylophilus

**Figure 3:**
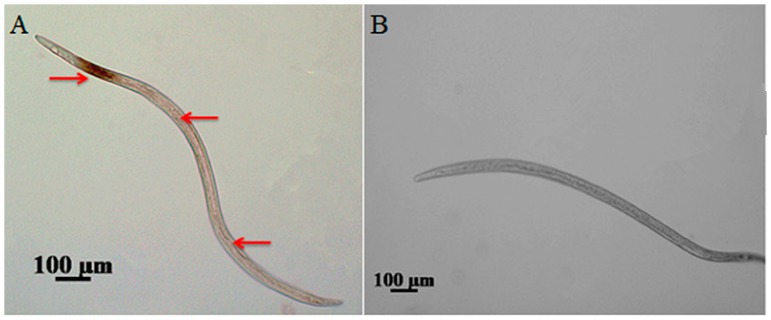
Localization of *Bx-ATG8* mRNA by *in situ* hybridization (ISH) using a digoxigenin (DIG)-labeled *BxATG8* antisense or sense probe. The hybridization sites are shown in *B. xylophilus* (**A**), including the oesophageal gland cells (right arrows), and lateral hypodermal seam cells (left arrows). Control sense probe in *B. xylophilus* (**B**). The scale bars = 100 μm.

An ISH method was used to analyze the subcellular localization of the autophagy gene *BxATG8*, and a digoxigenin (DIG)-labeled probe generated from *BxATG8* was specifically hybridized. The hybridization was observed in the oesophageal gland cells, as indicated by the dunk punctate color, and lateral hypodermal seam cells of *B. xylophilus*, as indicated by the light punctate color (Figure 3A). No hybridization was observed in the control group (Figure 3B). These results indicated that *BxATG8* was more highly expressed in oesophageal gland cells, which function to dilute abnormal proteins quickly and to recycle amino acids, thereby assisting *B. xylophilus* in invading its host. *BxATG8* was expressed in lateral hypodermal seam cells, which participate in metabolism and the storage of nutrients. We speculated that the function of autophagy in lateral hypodermal seam cells was regulating cellular metabolism and homeostasis.

### 2.4. Detection of RNAi Efficiency

**Figure 4:**
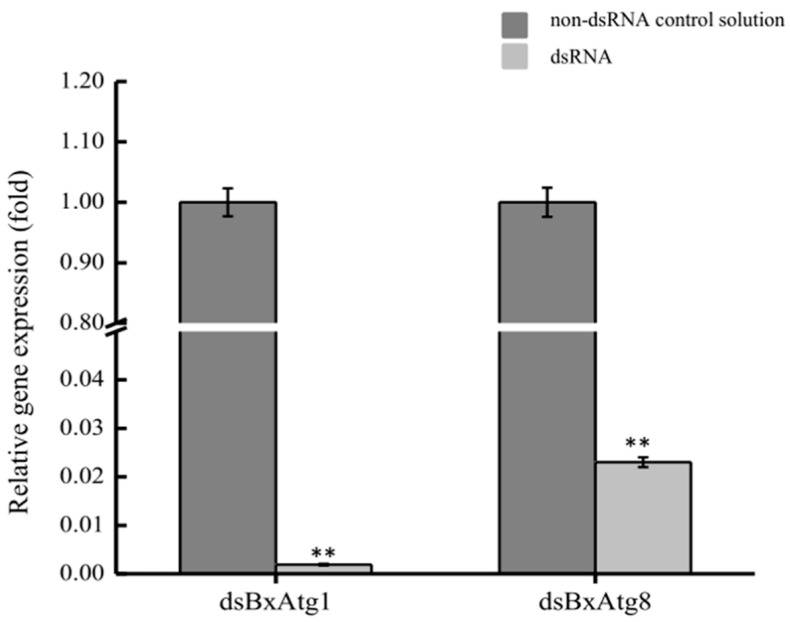
Quantitative reverse transcription PCR (qRT-PCR) analysis of the RNAi efficiency in *B. xylophilus* after treatment with ds*BxATG1* and ds*BxATG8*. *B. xylophilus* soaked in a non-dsRNA solution was used as the control. The expression level of the control was set as 100%. Data represent mean values ± standard deviation (SD) from three independent experiments. Bars show standard deviations of the mean. Asterisks on top of the bars indicate statistically significant differences (** *p* < 0.01, Student’s *t*-test).

RNAi was used to assess the functions of *BxATG1* and *BxATG8* in *B. xylophilus* in this study. Quantitative reverse transcription PCR (qRT-PCR) was performed to determine the effect of RNAi on the *BxATG1* and *BxATG8* mRNA levels. Soaking nematodes in ds*BxATG1* and ds*BxATG8* solutions resulted in a marked decrease in *BxATG1* and *BxATG8* gene expression levels compared with those of nematodes soaked in a non-dsRNA control solution. When the mRNA expression level of the control was considered as 100%, the mean expression level of ds*BxATG1*- and ds*BxATG8*-treated samples were 0.19% and 2.30%, respectively (Figure 4). These results suggested that *BxATG1* and *BxATG8* were silenced by RNAi effectively.

### 2.5. Effect of RNAi on B. xylophilus Reproduction on Fungal Mats

**Figure 5:**
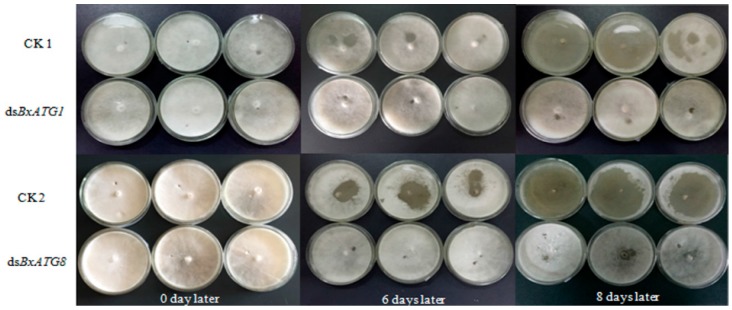
Effect of RNAi on *B. xylophilus* inoculated onto *Botrytis cinerea*. The nematodes soaked in ds*BxATG1*, ds*BxATG8* and non-dsRNA control solutions were grown in *B. cinerea*.

**Figure 6:**
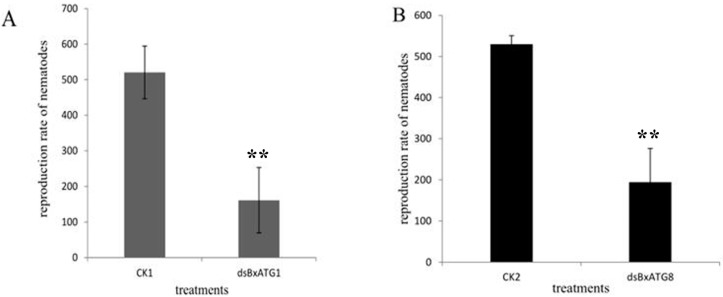
Effect of RNAi on *B. xylophilus* reproduction. Reproduction rates of *B. xylophilus* washed from potato dextrose agar (PDA) plates of *B. cinerea* with ds*BxATG1* and ds*BxATG8* and CK1 and CK2, respectively. Data represent mean values ± SD from three independent experiments. Bars show standard deviations of the mean. Asterisks on top of the bars indicate statistically significant differences between the ds*BxATG1* (**A**) and ds*BxATG8* (**B**) nematodes and controls (** *p* < 0.01, Student’s *t*-test).

The effect of RNAi on *B. xylophilus* reproduction was tested on potato dextrose agar (PDA) plates inoculated with *Botrytis cinerea* at 25 °C. The nematodes soaked in double-stranded RNA (ds*BxATG1* and ds*BxATG8*) solutions showed significantly reduced reproduction rates compared with the nematodes soaked in non-dsRNA control solutions (CK1 and CK2) and this result was confirmed by the remaining area of *B. cinerea* (Figure 5). After eight days, the reproduction rates of nematodes in the ds*BxATG1* and non-dsRNA control solution (CK1) treatment were 161- and 520-fold (*p* < 0.01, Student’s *t*-test) (Figure 6A), and the reproduction rates of nematodes in the ds*BxATG8* and non-dsRNA control solution (CK2) treatment were 194- and 529-fold (*p* < 0.01, Student’s *t*-test) (Figure 6B). These results indicated that *B. xylophilus* reproduction was significantly influenced by the RNAi treatment.

## 3. Discussion

Recently, a number of studies have focused on the functions of autophagy in eukaryotic organisms. The process of autophagy results in the turnover of intracellular proteins for guaranteed rejuvenation, which assists the clearance of misfolded proteins, and degrades organelles and proteins into small polypeptides to help maintain amino acid pools and the energy balance. Autophagy occurs constitutively at low levels even under normal growth conditions [17,27]. In this study, for the first time, the *B. xylophilus* autophagy was qualitatively identified during starvation, which is the best inducer of autophagy. It indicated that the process of autophagy exists in *B. xylophilus* as a response to stressful environmental conditions. The full-length cDNAs of autophagy-related genes (*BxATG1* and *BxATG8*) from *B. xylophilus*, which had never been reported previously, were cloned and analyzed. These findings are relevant given the central roles that their products play in the autophagy process.

ISH enables the investigation of gene expression patterns and gene functions in nematodes [28,29,30]. ATG8/LC3/LGG-1 is routinely used as a marker to study autophagy, and researchers rely heavily on the expression patterns of reporters for ATG8/LC3/LGG-1 [31,32,33]. Thus, ISH was used to locate *BxATG8* in *B. xylophilus* in this study, and the pattern of *BxATG8* in *B. xylophilus* showed that it was expressed in the oesophageal gland and lateral hypodermal seam cells. The oesophageal gland of nematodes secretes a large number of proteins, including glucanases and pectate lyase [34,35]. Both cellulase and pectate lyase proteins are secreted through the nematode stylet into plant tissues and participate in weakening the cell walls, which facilitates the feeding, penetration and migration of nematodes in pine tissues [35]. The roles of lateral hypodermal seam cells in *B. xylophilus* are in metabolism and storage of nutrients [36]. The role of autophagy is to quickly break down abnormal proteins and to recycle amino acids for combining proteins. The autophagic compartments are a continuous source of small peptides and amino acids used to rebuild cell structures [17]. Therefore, the results suggested that autophagy gene *BxATG8* might play an important role in plant–nematode interactions.

Furthermore, autophagy in pathogens, such as *Aedes aegypti*, *Magnaporthe oryzae* and *Colletotrichum orbiculare*, plays an important role in reproductive development, promoting their survival when environmental stress affects and changes their pathology [19,37,38]. Based on our results, we have found that these phenomena also occur in *B. xylophilus*. This was the first example of autophagy-related gene functions in *B. xylophilus*. RNAi technology was used to demonstrate the functions of *BxATG1* and *BxATG8*. RNAi was first described by Fire *et al.* [39]. Later, RNAi was developed as an effective tool in plants and animals to study gene functions and for genetic manipulation [40,41]. Moreover, RNAi has also been used to assess the pathogenic and molecular effects of silenced *B. xylophilus* genes [42,43,44,45]. Autophagy is believed to be associated with changes in cellular architecture during differentiation and development [14]. As in *Caenorhabditis elegans*, autophagy functions in the cellular processes that regulate life-span during non-stressed conditions, and *UNC-51* and *BEC-1* are required for male tail development. *C. elegans* failed to resume reproduction even under favorable environmental conditions when *BEC-1* was silenced [12,46]. Our results showed that the silencing of *BxATG1* and *BxATG8* reduced its reproductive capability. It demonstrated that *BxATG1* and *BxATG8* were necessary in the developmental processes of *B. xylophilus*. However, the potential role of autophagy in *B. xylophilus* needs to be further investigated.

## 4. Materials and Methods

### 4.1. B. xylophilus Growth Conditions and Experimental Organisms

The highly virulent AmA3 strain of *B. xylophilus* was isolated from wood chips of infested *Pinus thunbergii* Parl from Maanshan city, China. The virulence of AmA3 strain was evaluated by Xiang *et al.* [47]. The nematodes were grown in colonies of *B. cinerea* Pers, cultured on PDA plates for 7 days at 25 °C. Then, they were extracted overnight from PDA plates using the Baermann funnel method [48]. Two-year-old *P. thunbergii* seedlings were obtained from the greenhouse at Nanjing Forestry University (Nanjing, China).

### 4.2. TEM as Tool to Study Autophagy in B. xylophilus

The nematodes were subject to long-term starvation for 12, 24 and 36 h in double-distilled water (ddH_2_O). According to the TEM method [23,49], phosphate buffered saline (PBS) was made up of dibasic sodium phosphate and sodium dihydrogen phosphate (pH = 7.2). Nematodes were washed three times in PBS and placed in a 1.5 mL centrifuge tube with 4% glutaraldehyde fixative and fixed overnight at 4 °C, and then prepared for post-fixation in 2% osmium tetroxide (OsO_4_). The nematodes were placed in a graded series of acetone for 30 min each: 30%, 50%, 75%, 95%, and 2 × 100%. After adding 100% acetone, the centrifuge tube was capped to prevent moisture from entering. Acetone was mixed at ratios of 3:1, 1:1 and 1:3 with the Epon 812 resin mixture, and then added to the nematodes. Pure Epon 812 resin mixture was added to the nematodes, and one piece of the nematodes was placed into the bottom of each capsule. The capsules were placed into a 75 °C oven overnight to polymerize and were then cut into thin sections of 50–70 nm. Finally, the thin sections were stained and photographed under a TEM (JEM1400, Tokyo, Japan).

### 4.3. RNA Isolation and cDNA Synthesis of B. xylophilus

The total RNA of collected nematodes (a mixture of adults and juveniles) was extracted using TRIzol reagent (Invitrogen, Waltham, MA, USA). The RNA was quantified using a spectrophotometer and examined by electrophoresis on a 1% agarose gel. cDNA was synthesized from 2 µg of total RNA using the TransScript II One-Step gDNA Removal and cDNA Synthesis SuperMix according to the manufacturer’s instructions (TransGen Biotech, Beijing, China).

### 4.4. Homology-Based Cloning of Partial BxATG1 and BxATG8 Sequences from B. xylophilus

A homology-based cloning approach was used to clone the full-length cDNAs of two novel autophagy-related genes *BxATG1* and *BxATG8*. Two degenerate primer sets (*BxATG1* and *BxATG8*) were designed based on bioinformatics analyses [50]. The following primers were used: F-BxATG1 and R-BxATG1; and F-BxATG8 and R-BxATG8 (Table 1). The PCR conditions were 94 °C for 3 min followed by 30 cycles, each consisting of denaturation at 94 °C for 30 s, annealing at 55 °C for 30 s, and extension at 72 °C for 1 min. The final extension step was at 72 °C for 10 min.

### 4.5. Full-Length cDNA Cloning of BxATG1 and BxATG8 from B. xylophilus

The full-length *BxATG1* and *BxATG8* cDNA were obtained using the 3′-Full RACE CoreSet with PrimeScript™ RTase kit (TaKaRa Biotechnology, Dalian, China) and 5′-Full RACE Kit with TAP (TaKaRa Biotechnology). Gene-specific primers of *BxATG1*: GSP1-1 (3′-Full RACE first round of PCR) and GSP1-2 (3′-Full RACE second round of PCR), and GSP1-3 (5′-Full RACE first round of PCR), and GSP1-4 (5′-Full RACE second round of PCR) (Table 1). Gene-specific primers of *BxATG8*: GSP8-1 (3′-Full RACE first round of PCR) and GSP8-2 (3′-Full RACE first round of PCR), and GSP8-3 (5′-Full RACE first round of PCR) and GSP8-4 (5′-Full RACE second round of PCR) (Table 1). There were designed for 3′ and 5′ RACE amplification based on the two partial sequences of *BxATG1* and *BxATG8*, which were obtained from the homology-based cloning results. The cycling profiles used were as follows: a cycle at 94 °C for 3 min, followed by 30 cycles, each consisting of denaturation at 94 °C for 30 s, annealing at 55 °C for 30 s, and extension at 72 °C for 2 min. The final extension step was at 72 °C for 10 min.

### 4.6. Cloning and Sequencing of BxAtg1 and BxAtg8

The amplified PCR products were confirmed by electrophoresis on 1% agarose gels and purified according to the Gel Extraction Kit (Axygen, Hangzhou, China) instructions. They were then cloned into the *pEASY-T1* vector (TransGen Biotech, Beijing, China), which was used to transformed *Escherichia coli Trans1-T1* (*E. coli*) competent cells (TransGen Biotech). The *E. coli* was then incubated overnight at 37 °C on LB plates containing ampicillin. The positive transformants were analyzed by PCR using primers M13F(-47) and M13R(-48) (Table 1). Once the correct clone was identified, a fresh bacterial suspension was submitted to the Nanjing Genscript sequencing company (Nanjing, China) for sequence analysis. The open reading frames of the cDNA sequences of *BxATG1* and *BxATG8* were found using the ORF Finder tool (available online: http://www.ncbi.nlm.nih.gov/projects/gorf/).

### 4.7. ISH

ISH was used to evaluate the functions of the autophagy-related gene *BxATG8*. For ISH, the DNA fragment used as the probe was amplified from the full-length cDNA clones of *BxATG8* with a specific primer pairs, BxATG8-I-F and BxATG8-I-R (Table 1). The DIG-labeled sense random primer and anti-sense cDNA probes were synthesized from *BxATG8*’ PCR products. The nematodes were pre-treated before the post-hybridization washing step according to the manufacturer’s instructions. Hybridization and detection were performed with the DIG-High Prime DNA Labelling and Detection Starter Kit I (Roche Diagnostics, Mannheim, Germany), and finally examined using a Zeiss Axio Image M2 microscope (Zeiss MicroImaging GmbH, Oberkochen, Germany).

### 4.8. BxATG1 and BxATG8 Interference Using Double-Stranded RNA

Double-stranded RNA (dsRNA) was synthesized using the MEGscript RNAi Kit (Ambion Inc., Austin, TX, USA) with the primers BxATG1-T7I-F, BxATG1-I-R, BxATG1-I-F, BxATG1-T7I-R, BxATG8-T7I-F, BxATG8-I-R, BxATG8-I-F and BxATG8-T7I-R (Table 1). The RNAi soaking method was performed according to Urwin *et al.* [51]. Freshly cultured nematodes were soaked in dsRNA solution (800 ng/µL) and incubated at 180 rpm for 48 h at 20 °C. The nematodes soaked in the corresponding non-dsRNA solution were used as controls. Each treatment had three replicates. Samples from each treatment were washed thoroughly with ddH_2_O several times after soaking and then used for additional experiments.

### 4.9. Analysis of Reproduction of B. xylophilus after RNAi

The method of picking adult virgin female nematodes was modified by Wang *et al.* [52]. The eggs collected in the watch glass were covered with 2 mL of distilled water and incubated at 25 °C in the dark. The eggs took 25–32 h to hatch in water. Second-stage juveniles were picked and transferred onto a PDA plate with *B. cinerea* and cultured at 25 °C for one day. Then, female propagative four stage juveniles were collected under a stereo microscope (Zeiss MicroImaging GmbH) at 1 h intervals. The female and male nematodes were soaked in non-dsRNA solution and dsRNA solution, respectively. Fifteen pairs of female and male nematodes were picked and transferred onto a PDA plate with *B. cinerea* and cultured at 25 °C for 8 days. Three biological replicates were conducted. Subsequently, the nematodes were extracted from PDA plates using the Baermann funnel method and the nematodes were counted.

### 4.10. Quantitative Reverse Transcription PCR (qRT-PCR)

qRT-PCR was performed to determine the effect of RNAi on *BxATG1* and *BxATG8* mRNA levels. qRT-PCR was then carried out using SYBR Green Master Mix (Vazyme, Nanjing, China). The *Actin* gene of *B. xylophilus* was used as an internal control, with the primers listed in Table 1. Relative expression levels were determined using the ABI Prism 7500 software (Applied Biosystems, Foster City, CA, USA) and the 2^−ΔΔ*C*t^ method. qRT-PCR was conducted with three biological replicates and three technical replicates.

### 4.11. Statistical Analysis

All assays were performed in triplication. The results shown are the means and standard deviation (SD) of three independent experiments calculated using Microsoft Excel. The statistical significance was determined using SPSS Statistics 17.0 software (IBM China Company Ltd., Beijing, China) to perform the paired *t*-tests. Asterisks indicate statistically significant differences (** *p* < 0.01, Student’s *t*-test).

## 5. Conclusions

In summary, this study focused on the autophagy, which was identified by TEM under starvation, in *B. xylophilus*. Autophagy played a significant role in *B. xylophilus*’ resistance to an adverse starvation-inducing environment. The molecular characterization and functional analysis by ISH and RNAi of *BxATG1* and *BxATG8* from *B. xylophilus* indicated these autophagy genes are associated with development and reproduction. These discoveries regarding the relationship between autophagy and *B. xylophilus* helped us to understand the biological adaptation mechanism of *B. xylophilus* under adverse environments, and the functions of autophagy genes (*BxATG1* and *BxATG8*) in the process of PWD. The process of autophagy may serve as a survival mechanism in *B. xylophilus* and provides fundamental information for facilitating understanding of PWD.

## Figures and Tables

**Table 1:** Polymerase chain reaction (PCR) primers used in the study.

Name of Primers	Sequence (5′–3′)
F-BxATG1	GGGCCGAGCAGCTGGTNGTNTAYGT
R-BxATG1	CACAGCTCGATGGCGTGNYKRTACAT
F-BxATG8	ACTTTGAGAAGCGTCGTG
R-BxATG8	TGTGGAATGACATTGTTGAC
GSP1-1	TCTTGTGATGGCTCAACGAC
GSP1-2	CTCGTTCTCAAGAGCTGGCT
GSP1-3	TGGAGATGGCTGAAGAGTCG
GSP1-4	CGTTGAGCCATCACAAGACT
GSP8-1	GTCGTGCTGAAGGTGAGAAGAT
GSP8-2	TGAAGGTGAGAAGATCCGTCGCAAGT
GSP8-3	ATAAAGCTGTCCCATCGTGGTCGT
GSP8-4	TCAGAGGGAACCAGATACTT
BxATG1-T7I-F	TAATACGACTCACTATAGGGAAGGCAGAAATCGGACA
BxATG1-I-R	AATCGGCTCATGGAAAA
BxATG1-I-F	AAGGCAGAAATCGGACA
BxATG1-T7I-R	TAATACGACTCACTATAGGGAATCGGCTCATGGAAAA
BxATG8-T7I-F	TAATACGACTCACTATAGGGAACCCAAGTTTGAGACCT
BxATG8-I-R	CGAAAACACTACAATAAGA
BxATG8-I-F	AACCCAAGTTTGAGACCT
BxATG8-T7I-R	TAATACGACTCACTATAGGGCGAAAACACTACAATAAGA
Actin F	GCAACACGGAGTTCGTTGTAGA
Actin R	GTATCGTCACCAACTGGGATGA
qBxATG1-F	AGAGTGTTGGGTGAGGGA
qBxATG1-R	CTCGGCATTGGTACATTATA
qBxATG8-F	GTCAACGATGTCATTCCCCA
qBxATG8-R	AACTGATCACTCTTCGGCGG
M13F(-47)	CGCCAGGGTTTTCCCAGTCACGAC
M13R(-48)	AGCGGATAACAATTTCACACAGGA
